# Serum Total Antioxidant Capacity of Epileptic Children before and after Monotherapy with Sodium Valproate, Carbamazepine, and Phenobarbital

**Published:** 2018

**Authors:** Mahmoud Reza ASHRAFI, Reza AZIZI MALAMIRI, Sedigheh SHAMS, Neda RASHIDI RANJBAR, Sara EBRAHIMI NASRABADI, Mohammadtaghi HAGHI ASHTIANI, Nargess SALADJEGHEH, Varasteh VAKILI ZARCH

**Affiliations:** 1 Paediatrics Center of Excellence, Department of Paediatric Neurology, Children's Medical Centre, Tehran University of Medical Sciences, Tehran, Iran; 2 Department of Paediatric Neurology, Golestan Medical, Educational, and Research Center, Ahvaz Jundishapur University of Medical Sciences, Ahvaz, Iran; 3 Paediatrics Centre of Excellence, Department of Pathology, Children's Medical Centre, Tehran University of Medical Sciences, Tehran, Iran; 4 Clinical Laboratory, 22 Bahman Clinic, Social Security Organization, Tehran, Iran

**Keywords:** Total antioxidant capacity, Children, Epilepsy, Anticonvulsant, Oxidative stress

## Abstract

**Objectives:**

This case-control study was carried out to compare serum total antioxidant capacity (TAC) in the newly diagnosed children with epilepsy and that of a control group of healthy children at the same age and probable effects of antiepileptic drugs (AEDs) prescription on it.

**Materials & Methods:**

Overall, 130 participants (65 in each group) aged between 1 and 17 yr old were enrolled. The study was conducted in Children’s Medical Center, the Pediatrics Center of Excellence, Tehran, Iran in 2010. Serum TAC test was done for both control and patients group before AED therapy and after 3 months of monotherapy with sodium valproate, carbamazepine and phenobarbital in patients. Serum TAC values were measured based on Erel's method using an automated commercial kit. This method is based on the bleaching of the characteristic color of a more stable 2, 2’azinobis (3ethylbenzothiazoline6sulfonic acid) radical cation by antioxidants. The results were expressed in mmol Trolox equivalent/l.

**Results:**

Serum TAC values were significantly lower in the patients group before drug administration [mean (SD): 1.31 (0.19) mmol/L] in comparison with the control group [mean (SD): 1.46 (0.21) mmol/L] (*P*<0.001). In the patient's group, no differences were found in the serum TAC before and 3 months after AED monotherapy.

**Conclusion:**

Reduced serum TAC and an increased vulnerability to oxidative stress should be considered as an etiologic factor in the children with epilepsy.

## Introduction

Seizures and epileptic syndromes are common in children. 10.5 million children under 15 yr have active epilepsy. Seizures and epileptic syndromes have different mechanisms but recent studies had a new look to oxidative stress in pathogenesis of different epilepsies and other neurological disorders (1-3). 

Oxidative stress is a product of normal metabolism in the brain or abnormal insults to the brain, for example, brain hemorrhage. Brain tissue also produces a considerable amount of free oxygen and nitrogen radicals because of a large number of mitochondria. The brain also is vulnerable to free radical damage because of a considerable amount of oxidisable polyunsaturated fatty acids. (3)

Different antioxidative mechanisms work together to reduce oxidative stress in the brain. Nonenzymatic antioxidants including serum albumin, serum bilirubin and uric acid, vitamin E, and microelements (selenium) along with enzymatic antioxidative mechanisms including superoxide dismutase and glutathione peroxidase, are the main antioxidative mechanisms of the brain (4). 

Many antiepileptic drugs, including those widely used in epileptic patients, are metabolized to generate reactive metabolites having oxidative effects in the brain. Few studies have been performed in children to show the effects of anticonvulsants on the oxidative and antioxidative balance, in addition, in the majority of these studies, enzymatic antioxidants (glutathione peroxidase, superoxide dismutase, catalase, etc.) have been measured (5-8).

Serum concentrations of enzymatic antioxidants could be measured, but these measurements are time-consuming, expensive and require complex techniques, therefore, we carried out a study to measure serum total antioxidant capacity (TAC) with a more practical method in a group of children with newly diagnosed epilepsy before therapy and to compare this capacity by that of a group of age and sex-matched healthy controls. 

## Materials & Methods

**Study design**

We carried out this case-control study to compare serum TAC in children with newly diagnosed epilepsy (either outpatients or inpatients) and that of a group of sex- and age-matched healthy controls. The effect of anticonvulsant monotherapy on the serum TAC also was studied in children with epilepsy before and 3 months after anticonvulsant monotherapy. The study was conducted in Children’s Medical Center, the Pediatrics Center of Excellence, as a major referral pediatric hospital of Tehran, Iran in 2010. 

**Patients**

To be enrolled, patients had to be less than 17 yr old and had to meet the diagnostic criteria for idiopathic epilepsy. Seizures and epilepsies were classified according to the International League Against Epilepsy (9). A child Neurologist selected AED according to the seizure type and the patient’s age. Phenobarbital, sodium valproate, and carbamazepine were administered as monotherapy. In the first group, 15 patients (7 males and 8 females) were given phenobarbital monotherapy, in the second group, 26 patients (17 males and 9 females) were administered sodium valproate monotherapy, and in the third group, 24 patients (11 males and 13 females) received carbamazepine monotherapy. The patients who received anticonvulsants had been on the same drug at least 3 months and serum drug levels were at the therapeutic level. Excluded cases were those children who had poor compliance and had been on chronic medication other than anticonvulsants. The control group was composed of healthy children less than 17 yr seen in the Children's Medical Center for routine vaccination, elective minor surgeries and routine monitoring for growth and development. For each patient, the following information was obtained; medical history and clinical examination with special emphasis on the age, sex, type, and frequency of seizures. 

The Ethics Committee of the Tehran University of Medical Sciences approved the study (no. 86-02-30-5635). Informed consent was obtained from the parents of all subjects studied. The study protocol conforms to the ethical guidelines of the 1975 Declaration of Helsinki as reflected in a priori approval by the institution\'s human research committee.

**Blood sample collection**

Blood samples were drawn by venipuncture from the antecubital vein using a disposal plastic syringe through a 23gauge needle in the morning between 8:00 and 11:00 am. Subjects fasted for 8 h before sample collection. All children with epilepsy were at least 24 h seizure-free before the sampling time. Samples were transferred to the laboratory within 2 h for biochemical analysis. 

**Biochemical analyses**

Blood samples were centrifuged at 1500 × gr for 10 min, and the serum was stored at 70 C until the time of analysis. Serum total antioxidant capacity values were measured based on Erel's method with an automated commercial kit (RANDOX total antioxidant status, RANDOX Laboratories Ltd. UK). This method is based on the suppression of the blue-green color of 2, 2’-azinobis-3-ethylbenzothiazoline-6-sulfonic acid (ABTS) radical cation by antioxidants. The results were expressed in mmol Trolox equivalent/l (10). 

We also assessed serum albumin, total bilirubin, and uric acid as nonenzymatic antioxidants in each group. Albumin and uric acid were measured spectrophotometric method (using Bromocresol green and uricase reactant, respectively). Bilirubin was measured with dichloroaniline method. All spectrophotometric assays performed on Hitachi 717 autoanalyzer.

**Statistical analysis**

A significant difference in the serum total antioxidant capacity between two groups was expected (power=80%, α=0.05, β=0.2). All data were reported as the means (SD). Categorical variables were analyzed by the χ^2^-test. Continuous variables were analyzed by independent sample *t*-test, paired sample t-test, and One Way ANOVA on ranks with pairwise multiple comparison procedures (Dunn's Method) when appropriate. A *P*<0.05 was considered significant. A biostatistician blinded to study groups performed the analysis.

## Results

One hundred and thirty participants (65 in each group) aged 1 to 17 yr were enrolled in this study. Generalized tonic-clonic seizures consisted of 50 cases (77%) and other seizure types were 8 (12.3%) with complex partial seizures (CPS), 4(6.1%) with simple partial seizure (SPS) and 3 (4.6%) with atonic seizure. In the patient's group, 15 were given phenobarbital monotherapy, 26 were given sodium valproate monotherapy, and 24 received carbamazepine monotherapy. We found no significant differences between the groups regarding gender distribution or mean age (Table 1).

The serum levels of total antioxidant capacity and nonenzymatic antioxidant parameter of the participants before AED administration are shown in Table 2. TAC was significantly lower in the patients with newly diagnosed epilepsy than that of the controls (*P* <0.001). Serum albumin and uric acid levels were not significantly different in the patients from the control, but serum total bilirubin was significantly lower in the patients with newly diagnosed epilepsy than that of the controls (*P* <0.001).

Three months after AED administration serum TAC and nonenzymatic antioxidant parameter again were measured in two groups. The total antioxidant capacity was significantly lower in the patients with newly diagnosed epilepsy who treated with sodium valproate and carbamazepine than that of the controls (*P* <0.05). Total antioxidant capacity had no significant difference between controls and who received phenobarbital. Serum albumin had no significant difference between controls and patients who treated with anticonvulsants after 3 months. Serum total bilirubin and uric acid were significantly different in study groups (Table 3). There were also no significant differences in total antioxidant capacity of the patients, before and 3 months after anticonvulsant administration based on the treatment groups (Table 4).

## Discussion

Serum TAC was significantly lower in children with newly diagnosed epilepsy before anticonvulsant initiation than that of the controls. Three months after receiving anticonvulsant monotherapy, serum total antioxidant capacity was significantly lower in children who received sodium valproate monotherapy and Carbamazepine monotherapy than that of the controls.

Excessive productions of free oxygen and nitrogen radicals and decreased antioxidant capacity, which result in membrane lipid peroxidation, have been implicated in the pathophysiology of neuronal hyperexcitability in the brain and seizure recurrence. Brain is rich in polyunsaturated fatty acids and has a high rate of oxidative metabolism with low antioxidant defense mechanism. Total antioxidant capacity of the organism includes enzymatic and nonenzymatic endogenous antioxidant. These antioxidant systems scavenge free radicals of oxygen and nitrogen. Under certain conditions, the increase in oxidants and decrease in antioxidants cannot be prevented, and the oxidative/antioxidative balance shifts towards the oxidative status. Antiepileptic drugs can increase lipid peroxidation by modulating antioxidant capacity, leading to seizure recurrence and therapeutic failure (11-13). 

Results of our study were in agreement with previous studies (4, 5, 14-17). Total antioxidant capacity was significantly reduced in untreated groups of adults with epilepsy in comparison to controls. There was also a marked reduction in total antioxidant capacity in the sodium valproate monotherapy groups compared to the carbamazepine monotherapy group (14). Our results were in accordance with that`s results, however, after 3 months of phenobarbital monotherapy no difference was found between controls and treated children with epilepsy. Moreover, in our study, children who received carbamazepine and sodium valproate monotherapy had significantly reduced total antioxidant capacity, 3 months after treatment, comparing to controls.

The serum total antioxidant capacity levels were lower in the untreated group comparing to controls. However, no significant difference was found in the serum total antioxidant capacity of sodium valproate, carbamazepine or phenobarbital monotherapy group and that of the controls (5). However, after treatment, serum total antioxidant capacity was significantly lower in children who received sodium valproate or carbamazepine. Both monotherapy and polytherapy did not change oxidative stress parameters in epileptic patients (14).

**Table 1:** Baseline characteristics of the participants

	Controls (n=65)	pH (n=15)	VPA (n=26)	CBZ (n=24)
Age(yr)^a^^,^^b^	8.3 (3.8)	7.1 (3.2)	7.9 (3.3)	8.3 (3.6)
Sex (m/f)	39/26	7/8	17/9	11/13
Dosage, mg/kg/day	NA	5-7	35-40	20-25
Abnormal EEG	NA	8	12	10
Duration of treatment, months	NA	3	3	3

a; mean (SD).

b ; one-way ANOVA showed no significant difference between groups. n; number of participants. PH; phenobarbital monotherapy group, VPA; sodium valproate monotherapy group, CBZ; carbamazepine monotherapy group. NA; not available.

**Table 2:** Comparison of total antioxidant capacity and plasma level of nonenzymatic antioxidant parameter of the participants before anticonvulsant administration

Parameter^s^	Controls (n=65)	Patients (n=65)	*P * ^b^
TAC, mmol Trolox equivalent/l	1.46 (0.21)	1.31 (0.19)	<0.001
Albumin, mg/dl	5.22 (0.7)	5.1 (0.6)	0.199
Total bilirubin, mg/dl	0.51 (0.26)	0.29 (0.18)	<0.001
Uric acid, mg/dl	3.7 (1.17)	3.91 (1.16)	0.171

a; Data are given as mean (SD), TAC; Total antioxidant capacity, n; number of participants, b; normality test (Shapiro-Wilk) failed and *P* by Mann-Whitney Rank Sum Test.

**Table 3:** Comparison of total antioxidant capacity and nonenzymatic antioxidant parameter of the controls and patients, 3 months after anticonvulsant administration

Parameter^a^	Controls (n=65)	PH (n=15)	VPA (n=26)	CBZ (n=24)	* P value*
TAC, mmol Trolox equivalent/l	1.46 (0.21)*	1.39 (0.28)	1.24 (0.24)*	1.28 (0.19)*	<0.05
Albumin, mg/dl	5.22 (0.7)	5.14 (0.5)	4.74 (0.7)	4.69 (0.3)	No difference
Total bilirubin, mg/dl	0.51 (0.26)*	0.18 (0.11)*	0.36 (0.16)*	0.5 (0.01)	<0.05
Uric acid, mg/dl	3.7 (1.17)*	5.02 (0.47)*	3.61 (0.39)	2.3 (0.21)*	<0.05

a ; Data are given as mean (SD), TAC; Total antioxidant capacity, n; number of participants,

* ; *P*^b ^< 0.05 b; One Way ANOVA on ranks with Pairwise Multiple Comparison Procedures (Dunn's Method), PH; phenobarbital monotherapy group, VPA; sodium valproate monotherapy group, CBZ; carbamazepine monotherapy group

**Table 4:** Comparison of total antioxidant capacity of the patients, before and 3 months after anticonvulsant administration based on the treatment group

	Total antioxidant capacity^a^		
Anticonvulsant	Before treatment	3 months after treatment	*P* b
Phenobarbital (n=15)	1.34 (0.18)	1.39 (0.28)	0.272
sodium valproate (n=26)	1.3 (0.21)	1.24 (0.24)	0.191
Carbamazepine (n=24)	1.26 (0.16)	1.28 (0.19)	0.701

n; number of patients.

a ; mmol Trolox equivalent/l, mean (SD).

b ; *P *value with paired sample *t*test

We also measured serum albumin, serum total bilirubin and uric acid in our participants. These are well-known endogenous antioxidant molecules in the serum (10). Uric acid concentrations were higher in untreated adults with epilepsy. There were also high uric acid concentrations in patients who treated with sodium valproate monotherapy(4). In our study, uric acid concentrations were significantly higher in children treated with phenobarbital than to the controls and other treatment groups. We also found that in children who received carbamazepine, uric acid concentrations were significantly lower than to controls and sodium valproate group.

The serum uric acid concentrations were lower in the sodium valproate monotherapy group than that of the untreated group. The serum bilirubin concentration was higher in the untreated epileptics than that of the controls, and albumin was lower in the sodium valproate group than that of the controls (5). In our study, serum albumin had no difference between treatment groups and controls. Moreover, serum total bilirubin was significantly higher in the controls than to sodium valproate and phenobarbital groups. We have no idea for these inconsistencies between our results and previous studies, but dietary habits and age of study population could be an explanation for these differences.

Change in nonenzymatic antioxidant levels may be a protective mechanism against decreased total antioxidant capacity (5, 16). However, our results did not support this hypothesis and nonenzymatic antioxidants had no significant increased serum levels in our patients.

The results of our study must be interpreted in the face of certain limits. In this study, only serum TAC and nonenzymatic antioxidant levels of the subjects were evaluated and the assessment of lipid peroxidation as the main oxidative marker of the oxidative stress was not performed. Another limit of our study was that at the beginning of the study we lost some of our patients. Therefore, we decided to have a short follow up and we narrowed the study to a short period of 3 months after anticonvulsant administration. 

**In conclusion,** reduced serum total antioxidant capacity and an increased vulnerability to oxidative stress should be considered in the children with epilepsy and may have an etiologic role. Beneficial effect of nutritional supplements was reported in several studies. Further studies on this subject are needed, along with a search for new anticonvulsants with antioxidant properties. 

## Conflict of interest

The authors declare that there is no conflict of interest.
